# (*E*)-1-[4-(Methyl­sulfan­yl)phen­yl]-2-(2,3,4-trimeth­oxy­phen­yl)ethene

**DOI:** 10.1107/S1600536812036288

**Published:** 2012-08-25

**Authors:** Agnieszka Gielara-Korzańska, Tomasz Stefański, Artur Korzański, Stanisław Sobiak

**Affiliations:** a Poznań University of Medical Sciences, Faculty of Pharmacy, Chair and Department of Chemical Technology of Drugs, Grunwaldzka 6, 60-780 Poznań, Poland; b Adam Mickiewicz University, Faculty of Chemistry, Department of Crystallography, Grunwaldzka 6, 60-780 Poznań, Poland

## Abstract

In the title compound, C_18_H_20_O_3_S, the rings are almost coplanar [inter-ring dihedral angle = 6.6 (2)°]. In the crystal, weak C—H⋯O hydrogen bonds between the meth­oxy groups connect adjacent mol­ecules, giving chains which extend along [001].

## Related literature

For the synthesis, see: Cushman *et al.* (1991 ▶); Ulman *et al.* (1990 ▶). For the chemopreventive, cardioprotective and neuroprotective activity of the natural stilbene derivative *trans*-resveratrol (3,4′,5-trihy­droxy­stilbene), see: Goswami & Das (2009 ▶). For preclinical and clinical studies of its therapeutic action in cancer diseases, see: Bishayee *et al.* (2010 ▶); Kundu & Surh (2008) ▶; Rimando & Suh (2008 ▶). For the cancer prevention activity of other natural compounds with stilbene backbones, see: Saiko *et al.* (2008 ▶); Rimando & Suh (2008 ▶). For similar structures, see: Sopková-de Oliveira Santos *et al.* (2009 ▶). For bond-length data, see: Glusker *et al.* (1996 ▶).
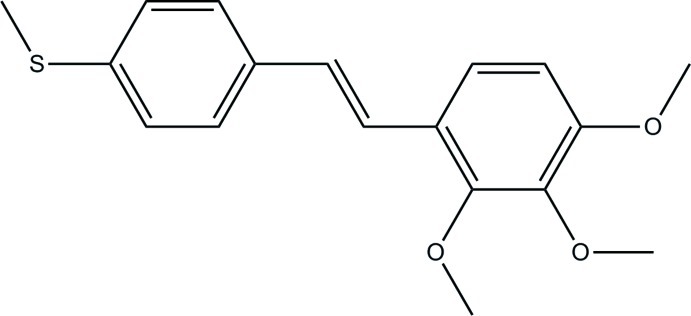


## Experimental

### 

#### Crystal data

C_18_H_20_O_3_S
*M* *_r_* = 316.40
Monoclinic, 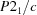

*a* = 13.9633 (4) Å
*b* = 7.7094 (2) Å
*c* = 15.1518 (4) Å
β = 90.705 (3)°
*V* = 1630.95 (8) Å^3^
*Z* = 4
Mo *K*α radiation
μ = 0.21 mm^−1^
*T* = 293 K
0.55 × 0.5 × 0.01 mm

#### Data collection

Agilent Xcalibur Atlas CCD-detector diffractometer
Absorption correction: multi-scan (*CrysAlis PRO*; Agilent, 2010 ▶) *T*_min_ = 0.900, *T*_max_ = 1.000
8931 measured reflections
2862 independent reflections
2229 reflections with *I* > 2σ(*I*)
*R*_int_ = 0.025

#### Refinement

*R*[*F* ^2^ > 2σ(*F* ^2^)] = 0.050
*wR*(*F* ^2^) = 0.105
*S* = 1.12
2862 reflections
279 parameters
All H-atom parameters refined
Δρ_max_ = 0.17 e Å^−3^
Δρ_min_ = −0.25 e Å^−3^

### 

Data collection: *CrysAlis PRO* (Agilent, 2010 ▶); cell refinement: *CrysAlis PRO*; data reduction: *CrysAlis PRO*; program(s) used to solve structure: *SHELXS97* (Sheldrick, 2008 ▶); program(s) used to refine structure: *SHELXL97* (Sheldrick, 2008 ▶); molecular graphics: *SHELXTL* (Sheldrick, 2008 ▶) and *Mercury* (Macrae *et al.*, 2008 ▶); software used to prepare material for publication: *SHELXL97*.

## Supplementary Material

Crystal structure: contains datablock(s) I, global. DOI: 10.1107/S1600536812036288/zs2220sup1.cif

Structure factors: contains datablock(s) I. DOI: 10.1107/S1600536812036288/zs2220Isup2.hkl

Supplementary material file. DOI: 10.1107/S1600536812036288/zs2220Isup3.cml

Additional supplementary materials:  crystallographic information; 3D view; checkCIF report

## Figures and Tables

**Table 1:** Hydrogen-bond geometry (Å, °)

*D*—H⋯*A*	*D*—H	H⋯*A*	*D*⋯*A*	*D*—H⋯*A*
C22—H22*B*⋯O17^i^	0.93 (3)	2.72 (3)	3.505 (4)	143 (2)

Symmetry code: (i) 
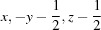
.

**Fig. 1.**
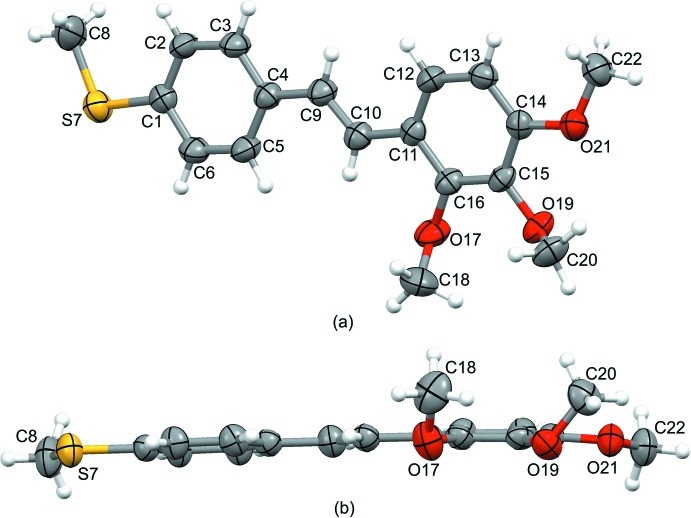
Mutually perpendicular views of the title compound [(a) and (b)], showing the atom-labelling scheme. Displacement ellipsoids are drawn at the 50% probability level.

**Fig. 2.**
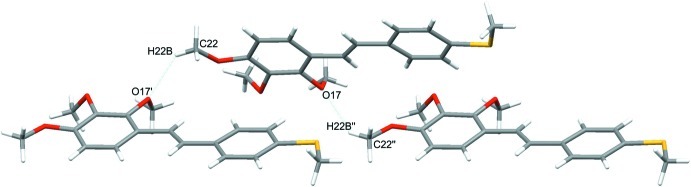
The hydrogen-bonded chain of molecules with weak intermolecular hydrogen bonds shown as dashed lines. For symmetry code (i), see Table 1. For symmetry code (ii): x, -y - 1/2, z + 1/2.

**Fig. 3.**
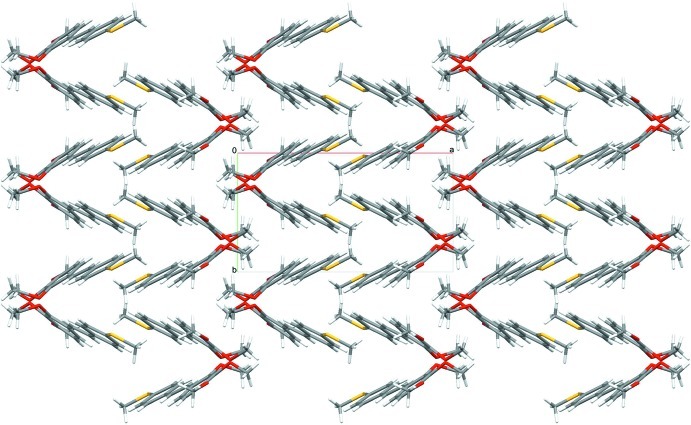
The crystal packing viewed down the chain direction [001].

## supplementary crystallographic information

### Comment

Chemopreventive, cardioprotective and neuroprotective activities of *trans*-resveratrol (3,4',5-trihydroxystilbene, RSV), the best known natural stilbene derivative, have been documented in numerous studies on animal models (Goswami & Das, 2009). Its therapeutic action in cancer diseases is also under intensive preclinical and clinical studies (Rimando & Suh, 2008; Bishayee *et al.*, 2010; Kundu & Surh, 2008). In the last decade, other natural compounds with stilbene backbones have been shown to possess promising cancer prevention activities (Saiko *et al.*, 2008; Rimando & Suh, 2008).

Interest in the concept and practice of chemoprevention as an approach to the control of cancer has increased especially due to the unsatisfactory results of classic chemotherapy. *In vitro* mechanisms of action of RSV have been extensively discussed in numerous reports and reviews. Several key mechanisms of action include: inhibition of the transcription factor NF-κ*B*, regulation of cytochrome P450 enzymes, activation of nuclear receptors such as estrogen receptors (ERs), inhibition of expression and activity of inflammation-related enzymes such as cyclooxygenases and regulation of sirtuins. These facts lead to the conclusion that RSV might be the potential lead structure for cancer chemopreventive and chemotherapeutic compounds.

Our previous studies have shown that a series of 4'-methylthio-*trans*-stilbene derivatives differing in the number and position of additional methoxy groups exhibited high affinity toward active sites of CYP1 enzymes involved in the activation of procarcinogens, in particular CYP1A1, CYP1A2 and CYP1B1. 2,3,4-Trimethoxy-4'-methylthio-*trans*-stilbene was found to be the most selective inhibitor of the enzymes CYP1A1 and CYP1B1 (IC_50_ values of 0.9 and 1.0 m*M* respectively, and exerted very low affinity to CYP1A2 (IC_50_ value above 50 m*M*).

In the title compound, C_18_H_20_O_3_S, (*E*)-1-(2,3,4-trimethoxyphenyl)-2-(4'-methylthiophenyl)ethene (Fig. 1*a*), the double bond C9—C10 in the conjugated linkage is in the *trans* configuration [torsion angle C(4)—C(9)—C(10)—C(11), 179.7 (2)°]. Furthermore, the value for the observed double bond [C9—C10, 1.319 (3) Å] is exactly as for the normal value (1.32 Å) and the single bonds [C(4)—C(9), 1.466 (3) Å and C(10)—C(11), 1.469 (3) Å] are shorter than the normal values (1.51 Å) (Glusker *et al.*, 1996), indicating the formation of a weak conjugated π-electron system. The aromatic rings do not deviate significantly from a coplanar arrangement, with a dihedral angle of 6.6 (2)° between the planes. Among the three methoxy substituents on the aromatic ring, only that at C14 is approximately coplanar with the benzene ring. The other two, at C15 and C16, are oriented towards opposite sides of the ring (Fig. 1 *b*) (Sopková-de Oliveira Santos *et al.*, 2009).

A very weak intermolecular contact is observed between the methoxy C22 – H22B group and atom O17^i^ of the methoxy group of a neighbouring molecule [for symmetry code: (i), see Table 1], giving one-dimensional chains which extend along [001] (Figs. 2, 3).

### Experimental

The key synthetic step for the construction of this compound involves the generation of diethyl 4-methylthiobenzyl phosphonate as an intermediate. This was prepared from commercially available 4-methylthiobenzyl alcohol in two steps. First, 4-methylthiobenzyl alcohol was converted to the chloride using SOCl_2_ in toluene at room temperature. Then, through the Michaelis-Arbuzov reaction of the 4-methylthiobenzyl chloride with triethylphosphite at 130 °C the corresponding phosphonate ester was obtained (Ulman *et al.*, 1990). The title compound was prepared by the Wittig-Horner reaction of diethyl 4-methylthiolbenzylphosphonate with the commercially available 2,3,4-trimethoxybenzaldehyde in DMF using sodium hydride as a base (Cushman *et al.*, 1991; Ulman *et al.*, 1990).

### Refinement

All hydrogen atoms were found in difference-Fourier maps and were freely refined with isotropic displacement parameters.

### Crystal data

**Table:**

C_18_H_20_O_3_S	*F*(000) = 672
*M**_r_* = 316.40	*D*_x_ = 1.289 Mg m^−^^3^
Monoclinic, *P*2_1_/*c*	Melting point: 417 K
Hall symbol: -P 2ybc	Mo *K*α radiation, λ = 0.71073 Å
*a* = 13.9633 (4) Å	Cell parameters from 6497 reflections
*b* = 7.7094 (2) Å	θ = 2.6–29.0°
*c* = 15.1518 (4) Å	µ = 0.21 mm^−^^1^
β = 90.705 (3)°	*T* = 293 K
*V* = 1630.95 (8) Å^3^	Plate, colourless
*Z* = 4	0.55 × 0.5 × 0.01 mm

### Data collection

**Table:**

Agilent Xcalibur Atlas CCD-detector diffractometer	2862 independent reflections
Radiation source: fine-focus sealed tube	2229 reflections with *I* > 2σ(*I*)
Graphite monochromator	*R*_int_ = 0.025
Detector resolution: 10.3088 pixels mm^-1^	θ_max_ = 25.0°, θ_min_ = 2.7°
ω scans	*h* = −14→16
Absorption correction: multi-scan (*CrysAlis PRO*; Agilent, 2010)	*k* = −9→8
*T*_min_ = 0.900, *T*_max_ = 1.000	*l* = −18→18
8931 measured reflections	

### Refinement

**Table:**

Refinement on *F*^2^	Primary atom site location: structure-invariant direct methods
Least-squares matrix: full	Secondary atom site location: difference Fourier map
*R*[*F*^2^ > 2σ(*F*^2^)] = 0.050	Hydrogen site location: difference Fourier map
*wR*(*F*^2^) = 0.105	All H-atom parameters refined
*S* = 1.12	*w* = 1/[σ^2^(*F*_o_^2^) + (0.0252*P*)^2^ + 1.2649*P*] where *P* = (*F*_o_^2^ + 2*F*_c_^2^)/3
2862 reflections	(Δ/σ)_max_ = 0.001
279 parameters	Δρ_max_ = 0.17 e Å^−^^3^
0 restraints	Δρ_min_ = −0.25 e Å^−^^3^

### Special details

**Table:**

Geometry. All e.s.d.'s (except the e.s.d. in the dihedral angle between two l.s. planes) are estimated using the full covariance matrix. The cell e.s.d.'s are taken into account individually in the estimation of e.s.d.'s in distances, angles and torsion angles; correlations between e.s.d.'s in cell parameters are only used when they are defined by crystal symmetry. An approximate (isotropic) treatment of cell e.s.d.'s is used for estimating e.s.d.'s involving l.s. planes.
Refinement. Refinement of *F*^2^ against ALL reflections. The weighted *R*-factor *wR* and goodness of fit *S* are based on *F*^2^, conventional *R*-factors *R* are based on *F*, with *F* set to zero for negative *F*^2^. The threshold expression of *F*^2^ > σ(*F*^2^) is used only for calculating *R*-factors(gt) *etc*. and is not relevant to the choice of reflections for refinement. *R*-factors based on *F*^2^ are statistically about twice as large as those based on *F*, and *R*- factors based on ALL data will be even larger.

### Fractional atomic coordinates and isotropic or equivalent isotropic displacement parameters (Å^2^)

**Table:**

	*x*	*y*	*z*	*U*_iso_*/*U*_eq_	
C1	0.61061 (17)	0.0320 (3)	0.76027 (15)	0.0414 (6)	
C2	0.55297 (19)	0.1096 (4)	0.69676 (16)	0.0473 (6)	
H2	0.4970 (17)	0.165 (3)	0.7110 (14)	0.045 (7)*	
C3	0.57983 (19)	0.1080 (4)	0.60942 (16)	0.0485 (7)	
H3	0.5392 (18)	0.162 (4)	0.5676 (16)	0.055 (8)*	
C4	0.66434 (17)	0.0313 (3)	0.58195 (15)	0.0414 (6)	
C5	0.72238 (19)	−0.0446 (4)	0.64716 (17)	0.0476 (6)	
H5	0.7822 (19)	−0.096 (3)	0.6326 (16)	0.054 (8)*	
C6	0.69588 (19)	−0.0444 (4)	0.73424 (16)	0.0478 (6)	
H6	0.7355 (19)	−0.101 (4)	0.7777 (17)	0.059 (8)*	
S7	0.58562 (5)	0.02516 (10)	0.87413 (4)	0.0535 (2)	
C8	0.4734 (3)	0.1316 (6)	0.8820 (2)	0.0704 (10)	
H8C	0.482 (3)	0.258 (6)	0.863 (3)	0.120 (15)*	
H8B	0.428 (2)	0.071 (4)	0.850 (2)	0.081 (11)*	
H8A	0.456 (2)	0.131 (4)	0.942 (2)	0.086 (10)*	
C9	0.68881 (19)	0.0317 (4)	0.48811 (16)	0.0467 (6)	
H9	0.6450 (19)	0.091 (4)	0.4513 (17)	0.059 (8)*	
C10	0.76519 (19)	−0.0373 (3)	0.45162 (16)	0.0431 (6)	
H10	0.8099 (18)	−0.093 (3)	0.4862 (16)	0.050 (7)*	
C11	0.78855 (16)	−0.0365 (3)	0.35739 (14)	0.0393 (5)	
C12	0.73453 (18)	0.0533 (3)	0.29456 (16)	0.0445 (6)	
H12	0.6803 (18)	0.120 (3)	0.3124 (16)	0.054 (7)*	
C13	0.75702 (18)	0.0514 (4)	0.20615 (16)	0.0452 (6)	
H13	0.7196 (17)	0.117 (3)	0.1650 (16)	0.049 (7)*	
C14	0.83398 (17)	−0.0449 (3)	0.17705 (14)	0.0410 (6)	
C15	0.89145 (16)	−0.1333 (3)	0.23833 (15)	0.0388 (6)	
C16	0.86901 (17)	−0.1269 (3)	0.32758 (15)	0.0395 (6)	
O17	0.92198 (13)	−0.2190 (3)	0.38833 (11)	0.0578 (5)	
C18	1.0193 (3)	−0.1635 (6)	0.4006 (3)	0.0774 (11)	
H18B	1.035 (3)	−0.201 (5)	0.457 (3)	0.116 (14)*	
H18A	1.056 (3)	−0.217 (6)	0.356 (3)	0.139 (18)*	
H18C	1.024 (4)	−0.033 (8)	0.399 (3)	0.19 (2)*	
O19	0.96529 (12)	−0.2384 (2)	0.21239 (11)	0.0502 (5)	
C20	1.0435 (2)	−0.1512 (5)	0.1699 (2)	0.0614 (8)	
H20C	1.092 (3)	−0.230 (5)	0.166 (2)	0.105 (13)*	
H20B	1.027 (2)	−0.119 (5)	0.109 (2)	0.104 (13)*	
H20A	1.062 (3)	−0.048 (7)	0.200 (3)	0.15 (2)*	
O21	0.85858 (12)	−0.0665 (2)	0.09066 (10)	0.0511 (5)	
C22	0.7946 (2)	0.0007 (5)	0.02544 (19)	0.0604 (8)	
H22C	0.790 (2)	0.128 (4)	0.0280 (17)	0.067 (9)*	
H22B	0.820 (2)	−0.030 (4)	−0.0289 (19)	0.066 (9)*	
H22A	0.729 (3)	−0.053 (4)	0.030 (2)	0.093 (11)*	

### Atomic displacement parameters (Å^2^)

**Table:**

	*U*^11^	*U*^22^	*U*^33^	*U*^12^	*U*^13^	*U*^23^
C1	0.0440 (14)	0.0393 (14)	0.0407 (12)	−0.0025 (12)	−0.0003 (10)	0.0012 (11)
C2	0.0412 (14)	0.0562 (17)	0.0445 (14)	0.0144 (13)	0.0042 (11)	0.0008 (12)
C3	0.0451 (15)	0.0568 (18)	0.0435 (14)	0.0140 (13)	−0.0012 (11)	0.0069 (12)
C4	0.0432 (13)	0.0376 (14)	0.0433 (12)	0.0018 (12)	0.0024 (10)	0.0016 (11)
C5	0.0430 (14)	0.0470 (16)	0.0527 (15)	0.0110 (13)	0.0044 (12)	0.0026 (12)
C6	0.0471 (15)	0.0502 (16)	0.0459 (14)	0.0097 (13)	−0.0036 (12)	0.0071 (12)
S7	0.0570 (4)	0.0646 (5)	0.0391 (3)	0.0026 (4)	0.0009 (3)	0.0045 (3)
C8	0.059 (2)	0.103 (3)	0.0489 (18)	0.006 (2)	0.0105 (15)	−0.0036 (19)
C9	0.0469 (15)	0.0486 (16)	0.0446 (13)	0.0087 (13)	0.0032 (11)	0.0044 (12)
C10	0.0463 (15)	0.0389 (14)	0.0441 (13)	0.0013 (12)	0.0035 (11)	0.0018 (11)
C11	0.0396 (13)	0.0352 (13)	0.0433 (12)	−0.0036 (11)	0.0030 (10)	−0.0026 (11)
C12	0.0392 (14)	0.0456 (16)	0.0487 (14)	0.0078 (12)	0.0029 (11)	−0.0028 (12)
C13	0.0415 (14)	0.0492 (16)	0.0447 (13)	0.0030 (13)	−0.0035 (11)	0.0027 (12)
C14	0.0412 (13)	0.0418 (14)	0.0401 (12)	−0.0064 (12)	0.0041 (10)	−0.0027 (11)
C15	0.0384 (13)	0.0288 (13)	0.0494 (13)	0.0004 (11)	0.0073 (10)	−0.0031 (10)
C16	0.0429 (13)	0.0305 (13)	0.0452 (13)	0.0010 (11)	0.0031 (10)	0.0035 (10)
O17	0.0595 (12)	0.0597 (13)	0.0545 (10)	0.0221 (10)	0.0079 (9)	0.0170 (9)
C18	0.070 (2)	0.090 (3)	0.072 (2)	0.023 (2)	−0.0233 (19)	−0.001 (2)
O19	0.0539 (11)	0.0388 (10)	0.0583 (10)	0.0096 (9)	0.0156 (8)	0.0020 (8)
C20	0.0468 (17)	0.064 (2)	0.074 (2)	0.0107 (17)	0.0185 (15)	0.0018 (18)
O21	0.0515 (10)	0.0631 (13)	0.0389 (9)	0.0022 (9)	0.0028 (8)	−0.0015 (8)
C22	0.065 (2)	0.073 (3)	0.0427 (15)	0.0063 (19)	−0.0016 (14)	0.0032 (15)

### Geometric parameters (Å, º)

**Table:**

C1—C2	1.383 (3)	C12—C13	1.380 (3)
C1—C6	1.390 (3)	C12—H12	0.96 (3)
C1—S7	1.765 (2)	C13—C14	1.382 (3)
C2—C3	1.380 (3)	C13—H13	0.95 (2)
C2—H2	0.92 (2)	C14—O21	1.368 (3)
C3—C4	1.388 (3)	C14—C15	1.397 (3)
C3—H3	0.94 (3)	C15—O19	1.373 (3)
C4—C5	1.398 (3)	C15—C16	1.393 (3)
C4—C9	1.466 (3)	C16—O17	1.372 (3)
C5—C6	1.375 (3)	O17—C18	1.435 (4)
C5—H5	0.95 (3)	C18—H18B	0.92 (4)
C6—H6	0.96 (3)	C18—H18A	0.95 (4)
S7—C8	1.774 (3)	C18—H18C	1.00 (6)
C8—H8C	1.03 (4)	O19—C20	1.441 (3)
C8—H8B	0.93 (3)	C20—H20C	0.91 (4)
C8—H8A	0.94 (3)	C20—H20B	0.98 (4)
C9—C10	1.319 (3)	C20—H20A	0.95 (5)
C9—H9	0.94 (3)	O21—C22	1.422 (3)
C10—C11	1.469 (3)	C22—H22C	0.99 (3)
C10—H10	0.92 (2)	C22—H22B	0.93 (3)
C11—C12	1.392 (3)	C22—H22A	1.01 (3)
C11—C16	1.401 (3)		
			
C2—C1—C6	118.6 (2)	C13—C12—H12	118.0 (15)
C2—C1—S7	124.92 (19)	C11—C12—H12	119.9 (15)
C6—C1—S7	116.43 (18)	C12—C13—C14	120.2 (2)
C3—C2—C1	120.0 (2)	C12—C13—H13	120.0 (15)
C3—C2—H2	118.2 (14)	C14—C13—H13	119.9 (15)
C1—C2—H2	121.8 (14)	O21—C14—C13	125.2 (2)
C2—C3—C4	122.2 (2)	O21—C14—C15	115.3 (2)
C2—C3—H3	118.2 (15)	C13—C14—C15	119.5 (2)
C4—C3—H3	119.6 (15)	O19—C15—C16	118.5 (2)
C3—C4—C5	117.0 (2)	O19—C15—C14	121.7 (2)
C3—C4—C9	119.9 (2)	C16—C15—C14	119.6 (2)
C5—C4—C9	123.1 (2)	O17—C16—C15	120.5 (2)
C6—C5—C4	121.2 (2)	O17—C16—C11	118.0 (2)
C6—C5—H5	117.9 (15)	C15—C16—C11	121.4 (2)
C4—C5—H5	120.9 (15)	C16—O17—C18	115.7 (2)
C5—C6—C1	121.0 (2)	O17—C18—H18B	104 (2)
C5—C6—H6	119.9 (16)	O17—C18—H18A	108 (3)
C1—C6—H6	119.1 (16)	H18B—C18—H18A	113 (4)
C1—S7—C8	103.76 (14)	O17—C18—H18C	111 (3)
S7—C8—H8C	108 (2)	H18B—C18—H18C	108 (4)
S7—C8—H8B	109 (2)	H18A—C18—H18C	113 (4)
H8C—C8—H8B	115 (3)	C15—O19—C20	115.4 (2)
S7—C8—H8A	108 (2)	O19—C20—H20C	107 (2)
H8C—C8—H8A	108 (3)	O19—C20—H20B	111 (2)
H8B—C8—H8A	109 (3)	H20C—C20—H20B	106 (3)
C10—C9—C4	127.2 (2)	O19—C20—H20A	112 (3)
C10—C9—H9	118.0 (16)	H20C—C20—H20A	112 (3)
C4—C9—H9	114.7 (16)	H20B—C20—H20A	108 (4)
C9—C10—C11	126.6 (2)	C14—O21—C22	117.2 (2)
C9—C10—H10	119.9 (15)	O21—C22—H22C	112.2 (17)
C11—C10—H10	113.5 (15)	O21—C22—H22B	106.2 (17)
C12—C11—C16	117.2 (2)	H22C—C22—H22B	108 (2)
C12—C11—C10	122.8 (2)	O21—C22—H22A	111.2 (19)
C16—C11—C10	120.0 (2)	H22C—C22—H22A	110 (3)
C13—C12—C11	122.1 (2)	H22B—C22—H22A	108 (3)
			
C6—C1—C2—C3	1.0 (4)	C12—C13—C14—O21	174.9 (2)
S7—C1—C2—C3	179.7 (2)	C12—C13—C14—C15	−3.1 (4)
C1—C2—C3—C4	−0.5 (4)	O21—C14—C15—O19	−2.0 (3)
C2—C3—C4—C5	−0.3 (4)	C13—C14—C15—O19	176.2 (2)
C2—C3—C4—C9	179.3 (3)	O21—C14—C15—C16	−176.6 (2)
C3—C4—C5—C6	0.7 (4)	C13—C14—C15—C16	1.6 (4)
C9—C4—C5—C6	−178.9 (3)	O19—C15—C16—O17	2.3 (3)
C4—C5—C6—C1	−0.3 (4)	C14—C15—C16—O17	177.1 (2)
C2—C1—C6—C5	−0.6 (4)	O19—C15—C16—C11	−173.4 (2)
S7—C1—C6—C5	−179.4 (2)	C14—C15—C16—C11	1.4 (4)
C2—C1—S7—C8	1.6 (3)	C12—C11—C16—O17	−178.7 (2)
C6—C1—S7—C8	−179.7 (2)	C10—C11—C16—O17	2.2 (3)
C3—C4—C9—C10	−179.0 (3)	C12—C11—C16—C15	−2.9 (4)
C5—C4—C9—C10	0.6 (4)	C10—C11—C16—C15	178.0 (2)
C4—C9—C10—C11	179.7 (2)	C15—C16—O17—C18	66.6 (3)
C9—C10—C11—C12	5.8 (4)	C11—C16—O17—C18	−117.6 (3)
C9—C10—C11—C16	−175.1 (3)	C16—C15—O19—C20	−119.7 (3)
C16—C11—C12—C13	1.5 (4)	C14—C15—O19—C20	65.6 (3)
C10—C11—C12—C13	−179.4 (2)	C13—C14—O21—C22	−6.6 (4)
C11—C12—C13—C14	1.5 (4)	C15—C14—O21—C22	171.5 (3)

### Hydrogen-bond geometry (Å, º)

**Table:**

*D*—H···*A*	*D*—H	H···*A*	*D*···*A*	*D*—H···*A*
C22—H22*B*···O17^i^	0.93 (3)	2.72 (3)	3.505 (4)	143 (2)

Symmetry code: (i) *x*, −*y*−1/2, *z*−1/2.
